# Cuticular hydrocarbon profiles reveal geographic chemotypes in stingless bees (Hymenoptera: Meliponini)

**DOI:** 10.1038/s41598-024-65298-5

**Published:** 2024-06-24

**Authors:** Melody Patricia Rodrigues Méndez, David Muñoz-Rodríguez, Rosendo Arturo de Jesús Aragón-Pech, José Octavio Macías Macías, José Javier G. Quezada-Euán

**Affiliations:** 1 Facultad de Ingeniería Química, UADY, Periférico Norte Km. 33.5, Colonia Chuburná de Hidalgo Inn, C.P. 97203 Mérida, Yucatán Mexico; 2 Departamento de Apicultura Tropical, Facultad de Medicina Veterinaria y Zootecnia, UADY, C.P. 97100 Mérida, Yucatán Mexico; 3 Centro Universitario de La Costa Sur, UDG, C.P. 48900 Autlán, Jalisco Mexico

**Keywords:** Chemical biology, Evolution, Zoology

## Abstract

Cuticular hydrocarbon (CHCs) variation has been detected in various insect taxa, but the potential contribution of cuticular compounds for analyzing intraspecific diversity at the population level has been little explored. Here we assess for the first time intraspecific variation in the CHC profile of stingless bees, using the species *Melipona beecheii* and *Nannotrigona perilampoides*. The objective is determining whether intraspecific variation can be useful for population identification. We found species-specific chemical patterns and extensive variation within each species. Notably, chemotypes were significantly associated to geographic origin in *N. perilampoides* but less so in *M. beecheii* and we discuss possible explanations for these patterns. Our results support the use of CHCs in conjunction with other methods in emerging problems such as undetected colony mobilization across regions. As CHCs are involved in several aspects of stingless bee recognition and interactions, it would be essential to unravel how these chemical signatures evolve across populations.

## Introduction

The composition and quantity of compounds on the surface of the cuticle of insects can be species-specific^1^. Thus, differences in cuticular profiles have been used as valid tools for species identification across several insect groups and for closely related ones^2^. As taxonomic tools, cuticular profiles are successfully combined with morphometrics and molecular tools for delimiting species^3,4^, including cryptic ones^5,6^. Notably, although chemotypes have been detected in various insect taxa^6–8^, the potential contribution of cuticular compounds for analyzing intraspecific diversity has been little explored.

The majority of insect cuticular compounds are hydrocarbons (CHCs), mainly alkanes, alkenes, and branched alkanes, with a chain length typically varying from C17 to C35, that are synthesized in specialized cells called oenocytes^9,10^. CHCs are subsequently transported (together with fatty acids, alcohols, glycerides, phospholipids and glycolipids) to the cuticle of the insect where they form a lipid wax layer that plays critical roles in the integrity and communication of individuals^1,11^. Primarily, CHCs serve as barrier to avoid water loss and abrasion of the insect’s body and protection against microorganisms^3,10^. In addition, in social insects CHCs can also convey information about the reproductive and physiological status, hierarchy, and task activity of different individuals^12–17^.

Most intraspecific-studies of CHCs in eusocial bees, including the Meliponini (Anthophila: Apidae) also known as stingless bees, have centered around differences among individuals and their role in communication. Thus, variation in cuticular profiles has been observed when comparing castes (queens and workers) in *Melipona bicolor*^18^ or among workers of different age and tasks groups in *Frieseomelitta varia*^19^, *Melipona marginata*^20^, *Friesella schrottkyi*^21^, *Tetragonisca angustula*^22^ and *Melipona solani*^23^. Although the importance of these compounds in the organization and function of colonies, is clear, considerably less is known about within species variation at the population level^3,24^.

Mesoamerica is a region where several stingless bee species have been cultured over centuries. Notably, the number of species of these insects under husbandry is increasing locally and worldwide^25^. Due to their popularity and escalating demand, translocation of stingless bee colonies across geographic regions is more frequently occurring^26,27^. Translocating colonies across regions or to regions naturally lacking stingless bees, can have negative consequences on the biodiversity and intraspecific genetic richness as well as in the spread of pathogens^27^. Accurate and rapid population delimitation thus, can be essential for conservation projects.

The primary objective of our study was to assess intraspecific variation in the CHCs profile of stingless bees, using two widely distributed species (*Melipona beecheii* and *Nannotrigona perilampoides*)^28–30^. Specifically, we aimed at characterizing the CHC profile of each species and to quantify differences among geographic populations that could allow identification for future conservation strategies.

## Methods

### Sample collection

Between February and July 2020, we sampled colonies of *M. beecheii* and *N. perilampoides*. Samples of foragers were mostly collected from colonies in meliponaries, i.e. traditional thatched roof structures for protection from rain and sunlight where stingless bee hives are kept. Only *N. perilampoides* collected from Jalisco had feral origin (Table 1). We collected foragers only, considering they would be within the same age and task category, as these traits influence the cuticular profiles in some species^19^.

**Table 1:** Number of colonies of *M. beecheii* and *N. perilampoides* sampled per Region, State, Locality and meliponary.

Species	Region	State	Locality	Number of colonies and meliponaries (in brackets)
*Melipona beecheii* *Nannotrigona perilampoides*	Yucatán Peninsula (PY)	Yucatán	Xmatkuil	3 (1)
			Mérida	4 (1)
			Umán	1 (1)
		Campeche	Calakmul	2 (2)
			Campeche	1 (1)
			Calkiní	6 (1)
		Quintana Roo	Felipe Carrillo	6 (1)
			Bacalar	4 (1)
	Tabasco (TAB East)	Tabasco	Emiliano Zapata	3 (1)
			Tenosique	3 (1)
	Tabasco (TAB West)		Tacotalpa	2 (1)
			Cunduacán	3 (2)
	Veracruz (VER)	Veracruz	San Juan Evangelista	3 (1)
			Jesús Carranza	2 (1)
			Coatepec	10 (1)
			Teocelo	3 (1)
	Puebla (PUE)	Puebla	Hueytamalco	7 (1)
			Teziutlán	2 (1)
	Oaxaca (OAX)	Oaxaca	San Juan Bautista	3 (1)
			Santiago Yaveo	2 (1)
			San Juan Guichicovi	7 (1)
	Chiapas (CHIA)	Chiapas	Viejo Montecristo	4 (1)
			Flor del Río	1 (1)
			San Antonio de los altos	2 (1)
	Total	**8**	**24**	**84**
	Yucatán Peninsula (PY)	Yucatán	Xmatkuil	3 (1)
		Campeche	Calkiní	4 (3)
		Quintana Roo	Felipe Carrillo	3 (1)
	Tabasco (TAB)	Tabasco	Tacotalpa	1 (1)
			Cunduacán	12 (2)
			Villahermosa	3 (1)
	Veracruz (VER)	Veracruz	San Juan Evangelista	3 (1)
			Jesús Carranza	3 (1)
			Soconusco	3 (1)
			Medellín de Bravo	4 (1)
			Comalteco	4 (2)
			Papantla	1
	Puebla (PUE)	Puebla	Hueytamalco	5 (2)
	Oaxaca (OAX)	Oaxaca	San Juan Bautista Valle	3 (1)
	Jalisco (JAL)	Jalisco	Zapopan	7*
	Total	**8**	**15**	**59**

*All colonies from this locality were feral.

Capital letters in brackets within Region denote the acronym used to refer to them in the text and in subsequent Tables and Figures.

Forager bees were collected with a sweep net at the colony entrance and killed at low temperature in an icebox (ca 0 °C). After this, both sets of wings from each specimen were dissected with a forceps and submerged in a glass vial containing 500 µl of hexane. We used wings because these are less prone to contamination from pollen, resin and other materials collected by foragers^31,32^. The same procedure was followed until the wings of five foragers from the same colony were submerged in the solvent. Each cuticular extract was labelled and kept in an ice box and later transferred to a freezer at − 20 °C until analysis.

**Figure 1:**
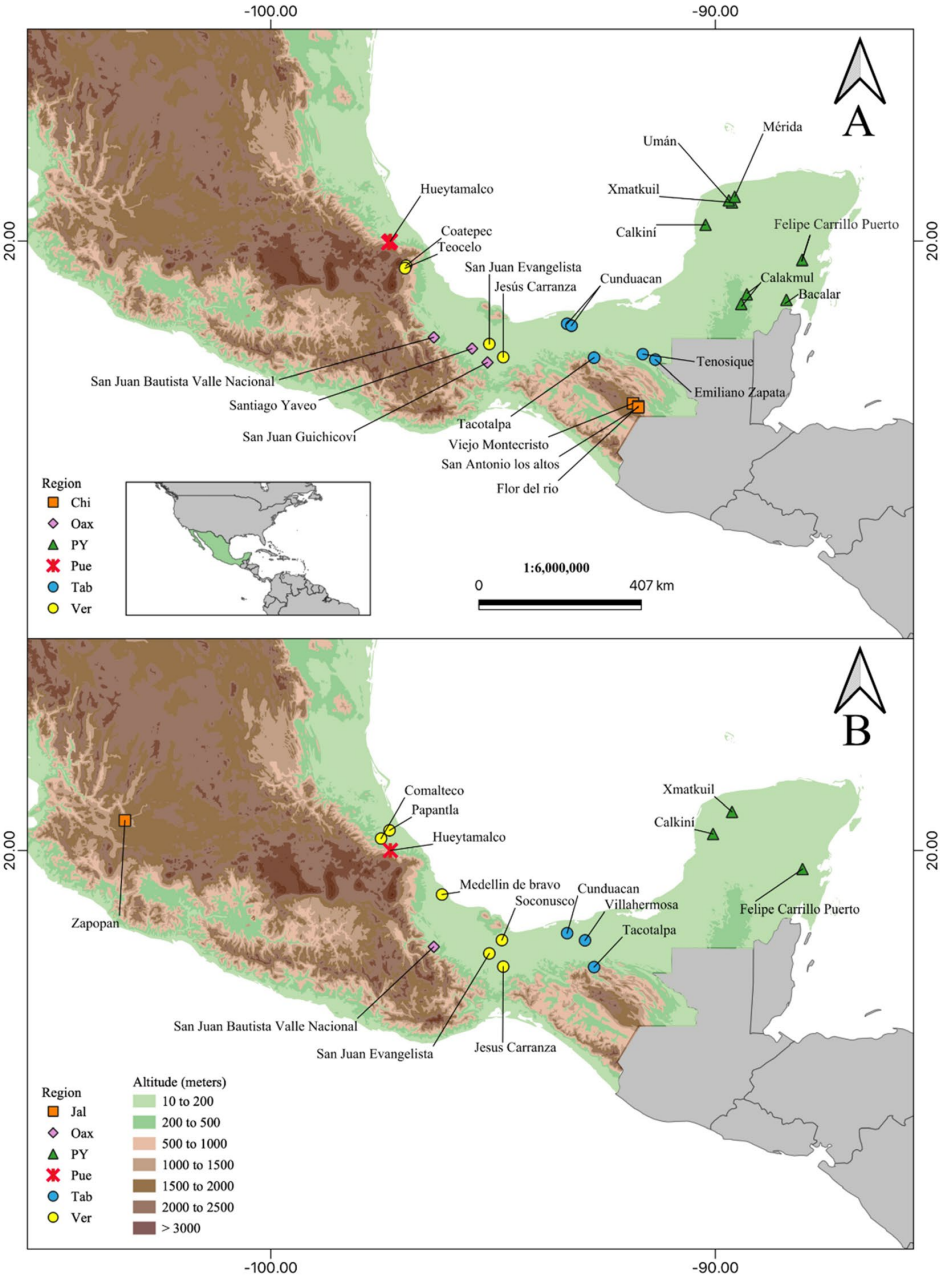
Map showing the localities where samples of *M. beecheii* (**A**) and *N. perilampoides* (**B**) were collected in different geographic regions across Mexico. Names of localities and acronyms for regions are those in Table 1.

A total of 84 colonies were sampled for *M. beecheii* and 59 for *N. perilampoides*, for which the same number of cuticular extracts were processed. The samples encompassed eight administrative states for both species and 24 localities for *M. beecheii* and 15 for *N. perilampoides* (Table 1; Fig. 1A and B).

### Chemical analysis of extracts

Extracts were analyzed on an Agilent Technologies 7890A gas chromatograph coupled to a 5975C Mass spectrometer. The separation was carried out with an Agilent HP-5MS capillary column (30 m × 250 µm × 0.25 µm). The oven was programmed with a temperature ramp from 40 to 240 °C at a speed of 40 °C/min, held for 5 min and then increased to 300 °C at 10 °C/min and held for 9 min. Helium was used as a carrier gas with a constant flow rate of 1 mL/min. For each analysis, 2 µL of wings extract was injected into the GC inlet at 300 °C in splitless mode (100 mL/min at 1.5 min). The mass spectrometer operated in electron impact mode at 70 eV. The temperature of the ion source was 230 °C and 150 °C for the quadrupole. A series of chromatograms were studied with the Agilent ChemStation software^33^ in which the compounds of the cuticular extracts were identified and characterized.

### Characterization and quantification of cuticular compounds

A standard mixture of C7 to C40 hydrocarbons (Merck, 49,452-U) with 5 µg/mL of each compound in hexane was analyzed to obtain their retention times and calculate the Kovats retention index of the unknown compounds. The retention times and indices obtained were subsequently compared with those reported in the literature or standard compounds for compound identification (see Supplementary files Table S1 online). The mass spectral patterns were also analyzed and compared to the NIST MS library^34^. The experimental Kovats retention indices (KI) were calculated with the formula:

$$I_{x} = 100n + \frac{{100\left( {t_{x} - t_{n} } \right)}}{{\left( {t_{n + 1} - t_{n} } \right)}}$$

The *n* symbolizes the number of carbons of the n-alkane that eluted before the unknown compound, t_x_ the retention time of the unknown compound, t_n_ the retention time of the n-alkane that eluted before t_x_ and t_n+1_ the time of the one that eluted immediately after t_x_.

The relative proportion of each identified CHC was calculated using the quotient of the sum of the peak areas of a given compound divided by the overall sum of the peak areas of all compounds for each locality. From the overall proportion of each compound, we calculated the proportion of alkanes and alkenes for each species. A general comparison of chemical profiles between *M. beecheii* and *N. perilampoides* was done by plotting the proportion of the main compound categories of CHCs, namely, alkanes, alkenes and their isomers. The relative proportion percentage (%) of each CHC in relation to the total composition of the extracts from each location was calculated. Comparison among geographic regions for each CHC per species were done by means of the Kruskal–Wallis statistic with the function ‘kruskal.test’^35^ followed by Dunn’s multiple comparison tests and the corrected Bonferroni values, using using the 'dunnTest' function of the ‘FSA’ package^36^.

### Comparison of chemical profiles

To compare the chemical profiles among geographic regions within each species, a non-metric multidimensional scaling (NMDS) analysis was performed using the ‘nmds’ function in R package ‘ecodist’^37,38^. The NMDS analysis was conducted using a triangular matrix generated as of the relative abundances. Based on the triangular matrix, we built a two-dimensional NMDS plot using 50 iterations per run in the R package ‘ecodist’^38^. To ensure the reproducibility of the NMDS plot, we used the ‘set.seed’ function^35^. In the NMDS plot, deviations are expressed in terms of “stress” where values below 0.15 indicate a good fit for the data^5^. Differences between regions were evaluated by one-way analysis of similarity (ANOSIM); both NMDS and ANOSIM were based on the Bray–Curtis dissimilarity index implemented in the R package ‘vegan’^39^. ANOSIM test is a special form of Mantel test, employed to determine whether there are significant differences between predetermined groups^4,5^. The generalized ANOSIM statistic *R* is defined as the slope of the linear regression of ranked resemblances from observations against ranked distances. The *R* values range from − 1 to 1, with values approaching 1 indicating complete dissimilarity between the groups^35^. A value of 0 implies no differences among the groups, whereas negative values are unusual and typically arise when there is significant dissimilarity within each group^40^.

Chemical dissimilarities among pairs of populations obtained after the ANOSIM for each species were used to build a matrix with the chemical distances for each species. The matrices of chemical distance and dissimilarity were then used to build a UPGMA dendrogram of distances among population using the program Dendro-UPGMA^41^. A matrix of geographical distances between populations was also calculated as the shortest distance connecting each pair of populations for each species. The matrices of chemical distance and geographical distance were submitted to a Mantel test using the ‘vegan package’^39^ of the software R software v.3.1.3^35^ to test for a correlation between chemical and geographical distances among populations (*P* value based on 100.000 resampling).

## Results

**Figure 2:**
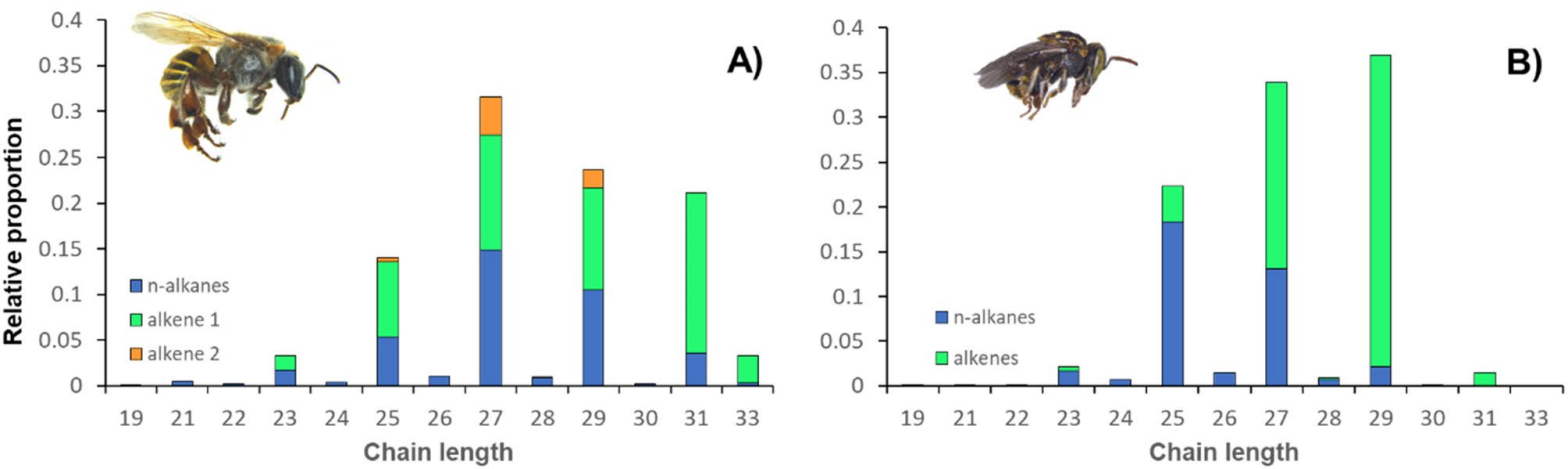
Relative proportion of cuticular hydrocarbons found in the stingless bees *Melipona beecheii* (**A**) and *Nannotrigona perilampoides* (**B**) from México*.*

The cuticular hydrocarbon profile of both studied species was contrasting. Despite sharing various hydrocarbons, the relative frequency of each compound significantly varied between species. It is also evident that the profile of *M. beecheii* included isomers of some alkenes, but in contrast *N. perilampoides* lacked isomers in its profile (Fig. 2). It is therefore, as expected, possible to differentiate both species in accordance to their general profile of cuticular hydrocarbons.

The chemical analysis of the cuticle of *M. beecheii* identified a total of 20 CHCs, three of them with isomers (Fig. 2A, Table 4). In the case of *N. perilampoides* the analysis of the cuticular profile identified a total of 16 CHCs, none of them with isomers (Fig. 2B, Table 5).

In Tables 2 and 3 we present the average relative proportion for the compounds found in the typical cuticular profile for colonies of *M. beecheii* and *N. perilampoides,* respectively, in each geographical location sampled.

**Table 2:** Relative proportion (± Standard error) of cuticular hydrocarbons found in *M. beecheii* for different states and regions of Mexico.

Compound	Average relative proportion (%) ± SE							H	*p-*value
	PY	TAB (East)	TAB (West)	VER	OAX	PUE	CHIA		
Nonadecane	0.11 ± 0.04 (41.11) ^a^	0.41 ± 0.10 (69.75) ^b^	0.00 ± 0.00 (29.50) ^a^	0.13 ± 0.04 (45.36) ^ab^	0.08 ± 0.04 (38.79) ^a^	0.13 ± 0.05 (45.06) ^ab^	0.00 ± 0.00 (29.50) ^a^	17.303	0.008
Heneicosane	1.18 ± 0.15 (65.67) ^c^	0.99 ± 0.18 (67.00) ^bc^	0.15 ± 0.09 (30.60) ^ab^	0.10 ± 0.03 (28.06) ^a^	0.19 ± 0.06 (33.50) ^ab^	0.00 ± 0.00 (19.00) ^a^	0.06 ± 0.06 (23.43) ^a^	57.047	0.001
Docosane	0.20 ± 0.08 (37.33) ^a^	0.00 ± 0.00 (24.00) ^a^	0.00 ± 0.00 (24.00) ^a^	0.20 ± 0.05 (41.78) ^ab^	0.37 ± 0.10 (52.67) ^ab^	0.71 ± 0.22 (65.33) ^b^	0.38 ± 0.20 (46.57) ^ab^	21.482	0.001
Tricosene	0.00 ± 0.00 (20.50) ^a^	0.00 ± 0.00 (20.50) ^a^	3.99 ± 0.97 (71.20) ^b^	3.30 ± 0.30 (69.44) ^b^	1.62 ± 0.27 (52.25) ^ab^	1.84 ± 0.08 (57.44) ^b^	0.00 ± 0.00 (20.50 ^a^)	74.790	0.001
Tricosane	0.23 ± 0.10 (19.24) ^a^	0.47 ± 0.06 (31.33) ^a^	6.74 ± 1.51 (74.80) ^d^	3.85 ± 0.25 (69.17) ^c^	1.78 ± 0.19 (49.00) ^b^	2.37 ± 0.12 (57.56) ^bc^	0.26 ± 0.22 (19.64) ^a^	68.543	0.001
Tetracosane	0.13 ± 0.08 (27.59) ^a^	0.00 ± 0.00 (22.00) ^a^	0.51 ± 0.17 (48.60) ^bc^	0.76 ± 0.15 (52.94) ^c^	0.7 ± 0.15 (53.42) ^c^	1.31 ± 0.22 (69.78) ^d^	0.22 ± 0.17 (32.57) ^b^	37.825	0.001
Pentacosene 1	2.06 ± 0.18 (19.56) ^a^	2.33 ± 0.08 (23.33) ^ab^	23.9 ± 3.67 (75.80) ^c^	17.79 ± 0.83 (70.61) ^c^	10.32 ± 1.56 (52.83) ^bc^	10.69 ± 0.28 (51.44) ^bc^	2.35 ± 0.47 (22.14) ^ab^	69.057	0.001
Pentacosene 2	0.09 ± 0.08 (33.93) ^a^	0.00 ± 0.00 (31.00) ^a^	0.00 ± 0.00 (31.00) ^a^	0.66 ± 0.26 (44.67) ^b^	0.05 ± 0.05 (33.67) ^a^	0.97 ± 0.13 (64.89) ^bc^	1.78 ± 0.31 (74.43) ^c^	43.871	0.001
Pentacosane	1.23 ± 0.35 (18.67) ^a^	0.73 ± 0.07 (18.33) ^a^	17.43 ± 2.72 (79.00) ^d^	10.37 ± 0.40 (71.00) ^c^	4.1 ± 0.44 (41.67) ^b^	7.49 ± 0.26 (56.89) ^bc^	3.33 ± 0.60 (38.71) ^b^	70.772	0.001
Hexacosane	0.43 ± 0.15 (24.76) ^a^	0.00 ± 0.00 (11.50) ^a^	1.09 ± 0.16 (50.20) ^ab^	1.78 ± 0.23 (60.28) ^b^	1.35 ± 0.23 (52.58) ^ab^	2.02 ± 0.32 (65.00) ^b^	1.35 ± 0.80 (40.07) ^ab^	44.610	0.001
Heptacosene 1	9.16 ± 0.71 (25.19) ^a^	9.1 ± 0.26 (27.67) ^a^	15.6 ± 2.14 (53.00) ^b^	19.81 ± 0.66 (68.94) ^c^	16.26 ± 1.77 (53.42) ^bc^	16.81 ± 0.53 (55.44) ^bc^	6.35 ± 0.65 (11.14) ^a^	54.411	0.001
Heptacosene 2	0.01 ± 0.01 (23.33) ^a^	0.00 ± 0.00 (22.50) ^a^	0.00 ± 0.00 (22.50) ^a^	5.03 ± 0.78 (54.97) ^b^	4.71 ± 0.85 (53.75) ^b^	4.87 ± 0.18 (54.56) ^b^	20.51 ± 1.94 (81.00) ^c^	59.510	0.001
Heptacosane	5.13 ± 0.82 (17.85) ^a^	5.93 ± 0.38 (22.67) ^a^	19.11 ± 3.55 (56.40) ^c^	18.12 ± 1.03 (58.61) ^c^	11.65 ± 0.63 (38.75) ^b^	19.53 ± 0.85 (64.78) ^c^	34.34 ± 1.50 (81.00) ^d^	66.243	0.001
Octacosene	0.00 ± 0.00 (31.00) ^a^	0.00 ± 0.00 (31.00) ^a^	0.00 ± 0.00 (31.00) ^a^	0.07 ± 0.03 (40.44) ^a^	0.32 ± 0.07 (63.33) ^b^	0.42 ± 0.10 (67.00) ^b^	0.11 ± 0.07 (43.00) ^ab^	42.797	0.001
Octacosane	0.55 ± 0.16 (28.67) ^a^	0.71 ± 0.27 (36.50) ^ab^	0.79 ± 0.25 (38.60) ^ab^	1.17 ± 0.14 (52.33) ^b^	1.23 ± 0.16 (53.50) ^b^	1.59 ± 0.23 (64.33) ^b^	0.88 ± 0.60 (31.57) ^ab^	23.472	0.001
Nonacosene 1	12.9 ± 0.86 (46.07) ^cd^	13.26 ± 0.31 (49.67) ^cd^	3.59 ± 1.68 (12.80) ^b^	9.48 ± 0.50 (29.17) ^c^	22.26 ± 1.08 (75.83) ^e^	14.12 ± 0.61 (55.00) ^d^	1.05 ± 0.55 (4.86) ^a^	55.333	0.001
Nonacosene 2	0.00 ± 0.00 (36.50) ^a^	0.00 ± 0.00 (36.50) ^a^	0.00 ± 0.00 (36.50) ^a^	0.00 ± 0.00 (36.50) ^a^	1.04 ± 0.55 (46.38) ^a^	0.35 ± 0.23 (44.72) ^a^	12.69 ± 1.42 (81.00) ^b^	57.263	0.001
Nonacosane	9.5 ± 0.41 (43.59) ^b^	17.87 ± 1.65 (79.00) ^c^	5.27 ± 1.96 (16.80) ^a^	6.61 ± 0.63 (22.00) ^a^	9.84 ± 1.25 (41.42) ^b^	10.8 ± 0.63 (53.11) ^b^	12.97 ± 0.65 (66.29) ^bc^	40.135	0.001
Triacontane	0.26 ± 0.08 (42.15) ^a^	0.15 ± 0.10 (39.33) ^a^	0.00 ± 0.00 (28.00) ^a^	0.13 ± 0.05 (38.17) ^a^	0.2 ± 0.09 (41.42) ^a^	0.5 ± 0.09 (62.89) ^a^	0.5 ± 0.36 (43.71) ^a^	12.192	0.057
Hentriacontene	40.73 ± 1.44 (69.85) ^d^	34.77 ± 1.84 (59.67) ^d^	0.32 ± 0.32 (15.20) ^ab^	0.40 ± 0.11 (17.44) ^ab^	10.19 ± 1.48 (44.67) ^c^	2.38 ± 0.21 (35.89) ^b^	0.00 ± 0.00 (11.00) ^a^	75.786	0.001
Hentriacontane	6.75 ± 0.40 (65.93) ^c^	8.68 ± 0.57 (75.00) ^c^	1.49 ± 0.78 (31.80) ^b^	0.22 ± 0.12 (15.83) ^a^	1.75 ± 0.47 (35.92) ^b^	1.09 ± 0.18 (32.56) ^b^	0.86 ± 0.46 (24.57) ^ab^	65.196	0.001
Tritriacontene	8.12 ± 0.64 (70.19) ^b^	4.23 ± 0.88 (58.17) ^b^	0.00 ± 0.00 (26.00) ^a^	0.00 ± 0.00 (26.00) ^a^	0.00 ± 0.00 (26.00) ^a^	0.00 ± 0.00 (26.00) ^a^	0.00 ± 0.00 (26.00) ^a^	78.057	0.001
Tritriacontane	1.14 ± 0.20 (60.67) ^b^	0.38 ± 0.17 (50.00) ^ab^	0.00 ± 0.00 (32.00) ^a^	0.00 ± 0.00 (32.00) ^a^	0.00 ± 0.00 (32.00) ^a^	0.00 ± 0.00 (32.00) ^a^	0.00 ± 0.00 (32.00) ^a^	43.228	0.001

The value of the Kruskal–Wallis test is represented by the letter H. The values in parenthesis represent the average ranks derived from Dunn’s multiple comparison test. Different letters denote significant differences at *p* < 0.05.

**Table 3:** Relative proportion (± Standard error) of cuticular hydrocarbons found in *N. perilampoides* for different states and regions of Mexico.

Compound	Average relative proportion (%) ± SE						H	*p*-value
	PY	TAB	VER	OAX	PUE	JAL		
Nonadecane	0.00 ± 0.00 (28.50)^a^	0.00 ± 0.00 (28.50)^a^	0.04 ± 0.03 (31.83)^a^	0.00 ± 0.00 (28.50)^a^	0.06 ± 0.06 (34.20)^a^	0.00 ± 0.00 (28.50)^a^	5.372	0.372
Heneicosane	0.00 ± 0.00 (26.50)^a^	0.03 ± 0.03 (28.16)^a^	0.07 ± 0.05 (29.94)^a^	0.36 ± 0.18 (45.17)^b^	0.23 ± 0.14 (38.90)^b^	0.00 ± 0.00 (26.50)^a^	14.5	0.013
Docosane	0.00 ± 0.00 (25.00)^a^	0.1 ± 0.05 (30.25)^a^	0.08 ± 0.05 (29.83)^a^	0.00 ± 0.00 (25.00)^a^	0.63 ± 0.23 (49.80)^b^	0.00 ± 0.00 (25.00)^a^	19.53	0.002
Tricosene	0.00 ± 0.00 (16.00)^a^	1.48 ± 0.33 (40.06)^bc^	0.12 ± 0.07 (19.61)^a^	1.11 ± 0.34 (41.33)^b^	0.42 ± 0.15 (31.00)^ab^	1.88 ± 0.31 (48.14)^c^	32.58	0.001
Tricosane	0.18 ± 0.12 (8.80)^a^	1.86 ± 0.23 (32.94)^b^	1.32 ± 0.21 (24.78)^b^	3.64 ± 0.24 (53.67)^c^	2.25 ± 0.40 (38.40)^bc^	3.65 ± 0.49 (50.86)^c^	34.81	0.001
Tetracosane	0.15 ± 0.11 (20.20)^a^	0.81 ± 0.19 (35.09)^ab^	0.78 ± 0.23 (32.06)^ab^	0.21 ± 0.21 (22.67)^ab^	2.16 ± 0.61 (50.60)^b^	0.00 ± 0.00 (15.50)^a^	20.32	0.001
Pentacosene 1	0.07 ± 0.07 (12.80)^a^	6.86 ± 0.68 (44.44)^c^	0.29 ± 0.12 (17.39)^a^	0.98 ± 0.47 (29.00)^b^	0.61 ± 0.05 (27.80)^ab^	40.9 ± 1.84 (56.00)^d^	49.39	0.001
Pentacosane	18.21 ± 1.86 (28.90)^ab^	15.75 ± 0.69 (19.13)^a^	18.73 ± 1.10 (29.89)^ab^	28.96 ± 2.17 (56.00)^b^	18.93 ± 1.67 (32.40)^ab^	22 ± 1.18 (43.86)^b^	17.99	0.003
Hexacosane	0.84 ± 0.36 (22.15)^ab^	1.3 ± 0.24 (30.72)^ab^	1.86 ± 0.37 (35.25)^b^	0.45 ± 0.06 (18.00)^a^	3.38 ± 0.96 (48.80)^b^	0.72 ± 0.56 (17.79)^a^	14.99	0.010
Heptacosene 1	13.74 ± 2.57 (15.40)^bc^	25.09 ± 0.78 (37.81)^c^	23.23 ± 1.84 (33.50)^c^	27.45 ± 1.18 (45.33)^c^	27.67 ± 2.88 (44.80)^c^	8.49 ± 0.84 (6.86)^a^	30.1	0.001
Heptacosane	14.47 ± 0.62 (44.50)^b^	9.6 ± 0.43 (19.69)^a^	15.33 ± 1.77 (40.11)^b^	13.35 ± 0.68 (41.33)^b^	9.54 ± 0.62 (19.80)^a^	7.70 ± 0.68 (9.29)^a^	32.38	0.001
Octacosene	0.00 ± 0.00 (21.50)^a^	0.36 ± 0.09 (38.09)^b^	0.24 ± 0.09 (31.89)^ab^	0.00 ± 0.00 (21.50)^a^	0.18 ± 0.12 (31.30)^ab^	0.00 ± 0.00 (21.50)^a^	13.6	0.018
Octacosane	0.12 ± 0.12 (19.70)^ab^	0.74 ± 0.19 (34.50)^b^	1.01 ± 0.30 (33.83)^c^	0.00 ± 0.00 (17.00)^a^	2.01 ± 0.68 (48.40)^d^	0.00 ± 0.00 (17.00)^a^	20.67	0.001
Nonacosene 1	50.44 ± 3.11 (51.70)^c^	36.01 ± 1.63 (36.50)^bc^	30.69 ± 2.15 (28.33)^b^	22.26 ± 0.32 (11.00)^ab^	24.63 ± 1.97 (17.20)^ab^	12.27 ± 2.27 (5.71)^a^	38.87	0.001
Nonacosane	1.78 ± 1.20 (24.90)^b^	0.00 ± 0.00 (16.50)^a^	3.67 ± 0.29 (46.78)^b^	0.34 ± 0.34 (22.00)^a^	2.43 ± 0.45 (39.60)^b^	0.65 ± 0.65 (21.57)^a^	37.88	0.001
Triacontane	0.00 ± 0.00 (29.00)^a^	0.00 ± 0.00 (29.00)^a^	0.05 ± 0.05 (30.61)^a^	0.00 ± 0.00 (29.00)^a^	0.26 ± 0.26 (35.00)^a^	0.00 ± 0.00 (29.00)^a^	5.784	0.328
Hentriacontene	0.00 ± 0.00 (19.00)^a^	0.00 ± 0.00 (19.00)^a^	2.5 ± 0.63 (37.39)^b^	0.89 ± 0.89 (27.33)^ab^	4.62 ± 0.27 (53.60)^b^	1.69 ± 0.52 (36.14)^ab^	32.39	0.001

The value of the Kruskal–Wallis test is represented by the letter H. The values in parenthesis represent the average ranks derived from Dunn’s multiple comparison test. Different letters denote significant differences at *p* < 0.05.

**Figure 3:**
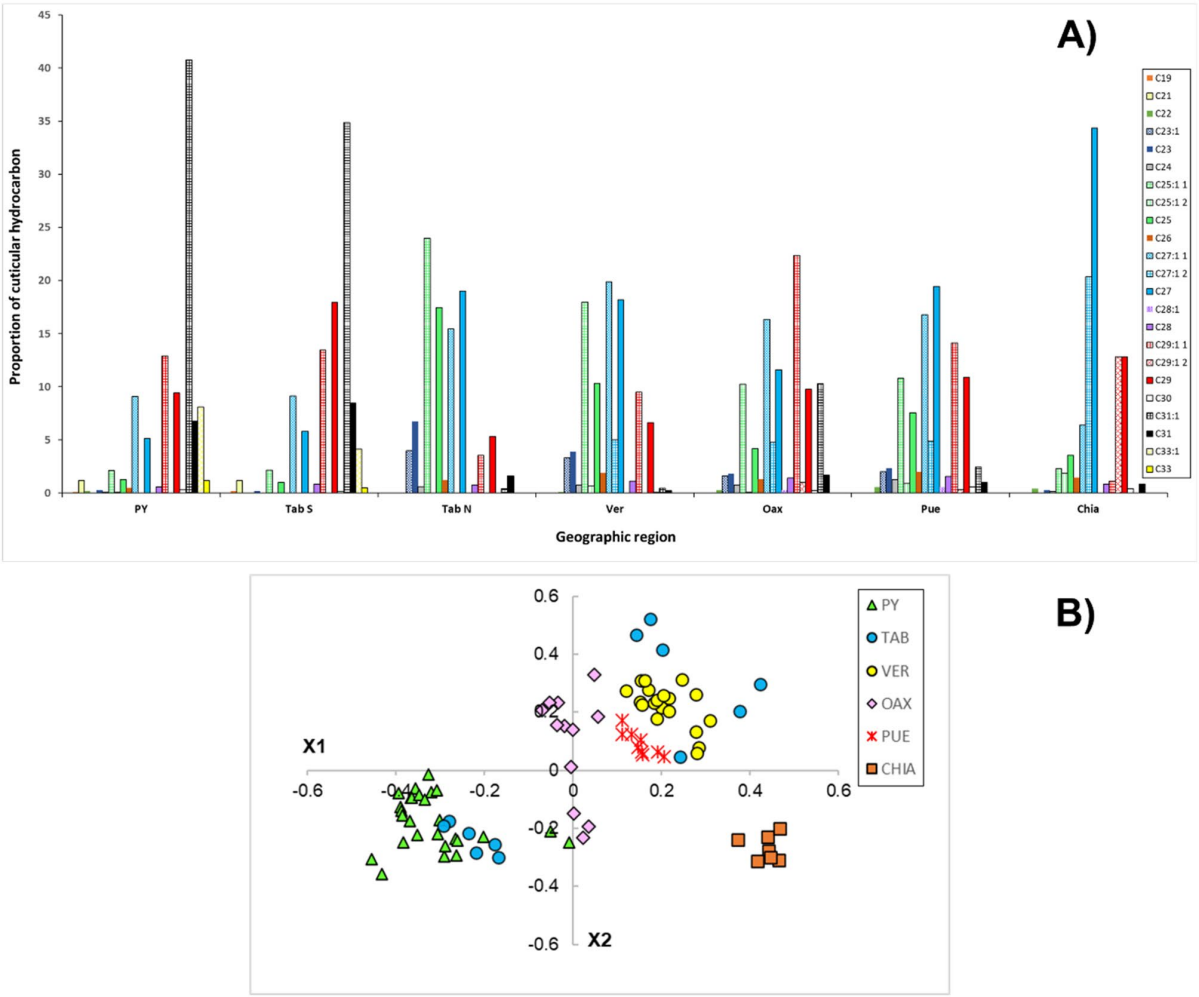
Relative frequencies of alkanes (solid bars) and alkenes (pattern bars) in the cuticular profile of *M. beecheii* colonies collected in different states and geographic regions across Mexico (**A**). In (**B**), Nonmetric multidimensional scaling (NMDS) plot of cuticular hydrocarbon variation among populations of *M. beecheii*. Stress value for NMDS configuration = 0.07. Acronyms are those in Table 1.

For *M. beecheii* the chain length of the identified CHCs varied between C19 and C33, which were classified as linear alkanes and alkenes. The most frequent CHCs in the populations studied were C25, C27, C29, C31 and C33 with their respective alkenes. Notably, the proportion of each of these CHCs varied across populations (Fig. 3A). The populations from PY and Tab East had less proportion of the alkane C25 and alkenes C25:1–1 and C25:1–2, as well as the alkane C27 and its alkenes C27:1–1 and C27:1–2, both of which were more frequent in the other four populations. In contrast, PY and Tab East had significantly higher proportion of C31 and its alkene C31:1 compared to the other regions. Likewise, alkane C33 was mostly detected in PY and Tab East compared with the other four regions where its presence was negligible (Fig. 3A). Thus, alkanes and alkenes of larger chain molecules were found in PY and Tab East, while CHCs with shorter chain lengths were found in the other four regions.

**Figure 4:**
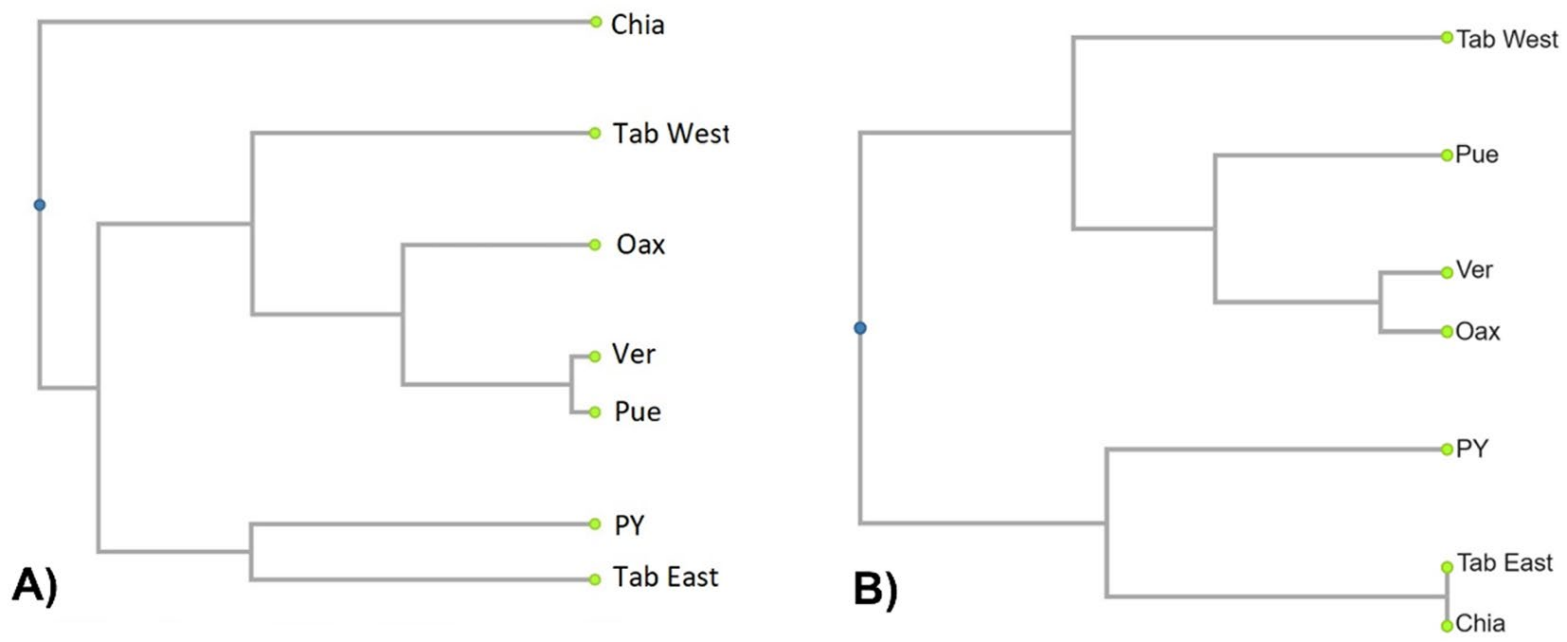
UPGMAs for *M. beecheii,* in (**A**) based on chemical distances and (**B**) based on geographic distance among different regions of Mexico. Acronyms are those in Table 1 and data are from Table 3.

In the case of *N. perilampoides*, the CHCs profile of this species varied between C21 and C31, which were also classified as linear alkanes and alkenes (Table 3). Overall, in *N. perilampoides*, it was more evident that the profile included a larger proportion of alkenes compared with alkanes, compared with *M. beecheii* (Fig. 4A). The most abundant CHC was the alkene C29:1 but its alkane was found in small proportion and in Tab was practically not detected. Other most frequent CHCs in the populations studied were C25 and C27 with their respective alkenes.

The plot of NMDS for *M. beecheii* showed clear separation among the different populations in accordance to their geographic origin (Fig. 3B). The results of the ANOSIM further supported this finding (Table 3). The overall value of the ANOSIM for *M. beecheii* was 0.859. The most distant populations regarding their hydrocarbon profiles were Tab East and Chia (ANOSIM = 1), followed by Tab West and Chia (ANOSIM = 0.978) and Tab East with Pue (ANOSIM = 0.975). Other ANOSIM values showing strong differences among paired populations (> 0.9) were those of Pue with Chia, PY with Tab West and the latter with Tab East. No significant differences were found between the paired populations of Tab West with Ver, Ver and Oax and Oax with Pue (this one had the lowest ANOSIM value = 0.038). The results of the UPGMA confirmed that it is possible to identify three main chemotypes among the studied populations of *M. beecheii* (Fig. 4A). One group formed by the bees from PY and East Tab, a second larger group formed by Tab West, Ver, Oax and Pue and finally a third group constituted solely by the bees of Chia alone. Some aspects worth to note are first, that the populations of *M. beecheii* from the state of Tabasco (Tab) can be clearly separated into two chemotypes, one from the East whose profiles are similar to the bees from PY and another of the Tab West bees which are more similar in their cuticular profiles to the bees from Ver, Pue and Oax. Second, the population of Chia forms a very compact and clearly separated group from all others. Lastly, two colonies from PY were placed close to the population from Oax, this is interesting, and it would be important to verify the origin and the possibility of colony exchange among these regions.

**Figure 5:**
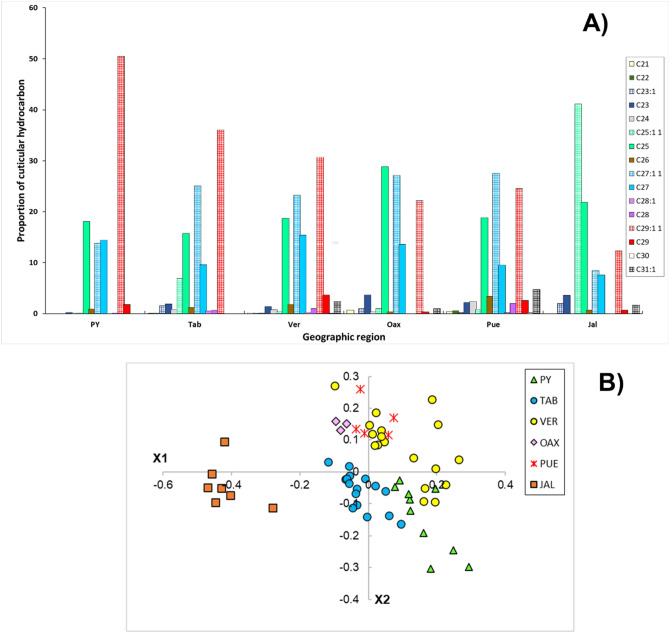
Relative frequencies of alkanes (solid bars) and alkenes (pattern bars) in the cuticular profile of *N. perilampoides* colonies collected in different states and geographic regions across Mexico (**A**). In (**B**), Nonmetric multidimensional scaling (NMDS) plot of cuticular hydrocarbon variation among populations of *N. perilampoides*. Stress value for NMDS configuration = 0.14. Acronyms are those in Table 1.

Similar to *M. beecheii*, the proportion of the different CHCs varied across populations of *N. perilampoides* (Fig. 5A). The populations from PY and Tab had the highest proportion of the alkene C29:1. Interestingly, this alkene seems to have a decreasing proportional gradient from the populations of East of Mexico towards the West, with the population on the Pacific state of Jal having the lowest proportion of this compound. A reverse situation seems to occur with alkane C25 which contributed proportionally less to the profile of populations in PY and Tab compared with the other four (Fig. 5A). Notably, the more frequent compound found in the profile of *N. perilampoides* from Jal was the alkene C25:1. Thus, alkenes of larger chain molecules were also found in *N. perilampoides* from PY and Tab, while compounds with shorter chain molecules were found in the other four regions.

**Figure 6:**
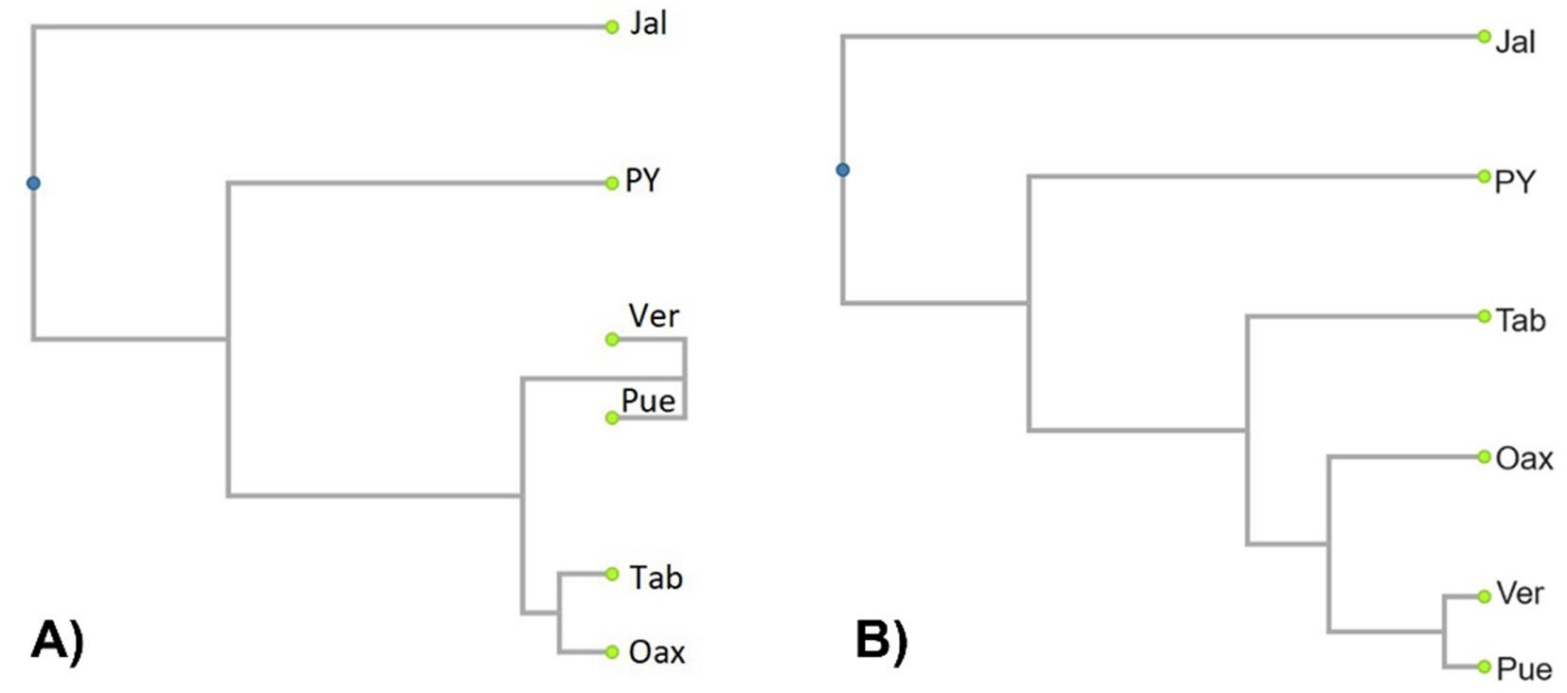
UPGMAs for *N. perilampoides,* in (**A**) based on chemical distances and (**B**) based on geographic distance among different regions of Mexico. Acronyms are those in Table 1 and data are from Table 3.

The NMDS plot for *N. perilampoides* showed separation among the different populations in accordance to their geographic origin (Fig. 5B). However, the value of the overall ANOSIM for this species was comparatively lower = 0.603 (Table 4), meaning that the separation among some populations was less pronounced. The most distant populations of *N. perilampoides* were Pue and Jal (ANOSIM = 0.996), followed by Jal and Oax (ANOSIM = 0.96) and Jal with PY (ANOSIM = 0.933). Other ANOSIM value showing strong differences among paired populations (= 0.868) was that of Pue with PY. Notably, six comparisons among paired populations showed no significant differences for *N. perilampoides*. No significant differences were found between Tab with Ver, Tab and Oax, Tab and Pue, Ver and Oax, Oax with Pue and Ver with Pue (this paired comparison had the lowest ANOSIM value =  − 0.102). Given these results, it is also possible to identify three main chemotypes in the UPGMA of the studied populations of *N. perilampoides* (Fig. 6). Clearly different to all regions is the population from Jal on the Pacific Coast, as well as the PY population which was significantly different to all others. A third larger group is formed by Tab, Ver, Oax and Pue (Fig. 6A). One important aspect to note is the negative value obtained for the paired comparison of Ver and Pue (Table 4 and Fig. 6A). This pattern usually results when the difference within one population is larger than the difference between populations^42^. In fact, on the NDSM plot it is possible noticing that while some colonies from Ver are similar to Pue and Oax, some colonies from Ver cluster separately from the former (Fig. 5B).

**Table 4:** Values for paired comparison of chemical distance of *M. beecheii* colonies from different geographic regions (values closer to 1 indicate larger differentiation).

	PY	Tab East	Tab West	Ver	Oax	Pue	Chia
PY		0.563	0.902	0.748	0.442	0.659	0.863
Tab East	395		0.92	0.702	0.876	0.975	1
Tab West	449	150		***0.111***	0.749	0.631	0.978
Ver	608	447	209		***0.259***	***0.038***	0.534
Oax	807	507	330.5	71.5		***0.37***	0.858
Pue	1136	843	666	128	371		0.941
Chia	337	330	355	586	531	975	

The global value of ANOSIM for all groups was R = 0.859. Figures in bold and italic indicate non-significant differences between populations (*p* > 0.01). Below the diagonal geographic distance between regions given in km. Acronyms for regions are those in Table 1.

The results of the Mantel test correlating chemical with geographic distance were highly significant in *N. perilampoides* $\left( {r_{M} = 0.804, P = 0.006} \right)$, as can be also seen on Fig. 6A and B. This result indicates isolation by distance as a possible factor causing differentiation of the populations in *N. perilampoides*. However, the value of the Mantel test was not significant in *M. beecheii* $\left( {r_{M} = 0.33, P = 0.094} \right)$, indicating that geographic separation is a comparatively less powerful factor in the differentiation among populations of this species (Table 5).

**Table 5:** Values for paired comparison of chemical distance of *N. perilampoides* colonies from different geographic regions (values closer to 1 indicate larger differentiation).

	PY	Tab	Ver	Oax	Pue	Jal
PY		0.408	0.405	0.48	0.868	0.933
Tab	472.2		***0.101***	***0.075***	***0.066***	0.574
Ver	701	212		***0.025***	***− 0.102***	0.611
Oax	856	365	173		***0.316***	0.96
Pue	1156	666	70	371		0.996
Jal	1769	1279.5	777	984.3	800.2	

The global value of ANOSIM for all groups was R = 0.603. Figures in bold and italic indicate non-significant differences between populations (*p* > 0.01). Below the diagonal geographic distance between regions given in km. Acronyms for regions are those in Table 1.

## Discussion

In general, there is still a lack of solid evidence on the natural intra-species variation in CHC profiles, even in well-established insect species^3^ and these compounds have thus, not been extensively used for chemotaxonomy in social hymenopterans^43^. We analyzed the profile of CHCs of the Mesoamerican stingless bees *M. beecheii* and *N. perilampoides* and found clear species-specific chemical patterns. Our results revealed extensive variation within each species and notably, that chemotypes were significantly linked to the geographic regions from where populations originated. This evidence supports the use of CHCs as additional markers for the study of variation and evolution in stingless bees.

Normally, CHCs vary between species in the types of compounds that they produce and/or the relative quantities of shared compounds^3,4^. Intraspecific CHC variation in the closely related ants and wasps is mostly quantitative, that is, individuals possess the same set of hydrocarbons, but in different relative quantities^44,45^. Likewise, our study in stingless bees revealed that in the two species studied, variation in the cuticular profiles could be found between species but also within species. Variation was mostly quantitative, but the profiles obtained even for similar CHCs was sufficient to clearly separate both species and to identify geographic populations within them.

Noteworthy, chemotypes were detected within each species and these were significantly determined by the geographic region. We found that alkanes with shorter chain lengths (molecules with a comparatively higher fluidity) were more frequent in *N. perilampoides* compared with *M. beecheii*. Moreover, more compounds were detected for both species in the regions with more constant humidity all year round compared with the Yucatan Peninsula which experiences a period of drought between March and May. Indeed, the Yucatán Peninsula is a separate biogeographic region with a distinct climate, predominantly of the Aw type with comparatively less rainfall (subhumid). In contrast, Veracruz and Tabasco are predominantly included in the Af and Am types, characterized by average higher rainfall^46^. Thus, the particular profile seems to be in concordance to the habitats in which both species are naturally present. Warm and dry conditions can lead to higher proportions of more aggregating substances like *n*-alkanes, while the more fluid unsaturated hydrocarbons tend to decrease^45^. *N. perilampoides* is a bee that can be found from altitudes at sea level up to 1500 m, even reaching cloud tropical forests where temperatures can be relatively low while *M. beecheii* is usually found at sea level not exceeding a couple hundred meters of altitude^30^. Even more, the dry conditions of the lowlands of Yucatan seem to also affect the pattern of CHCs with less diverse compounds found compared with the other regions. Such differences in habitat preference by both species and of the geographic region where their populations are found could be determinant in the pattern of cuticular hydrocarbons found in each.

Compounds within each species were identified that could be useful in the identification of geographic populations. In the case of *M. beecheii*, the alkene C31:1 was present in frequencies of 35% and above in the Yucatan Peninsula and Tab East. This compound was only marginal present in all the other populations, except in Oaxaca where it was present but with a frequency below 15%. Likewise, the frequency of C25 and C27 and its respective alkenes, were only marginal in populations from the Yucatan Peninsula and Tabasco East, but it was more readily identified in the other four populations, with the exception of Chiapas where C25 was also present at low frequency. Noteworthy, the frequency of C27 and C27:1 was highest in Chiapas where both represented more than 50% of the cuticular profile. In the case of *N. perilampoides* the alkene C29:1 was present at frequencies of 35% and above in populations from the Yucatan Peninsula and Tabasco, but below these levels in all other populations. The alkane C25 was also more frequent in populations other than the Yucatan Peninsula and Tabasco where its frequency was below 20%. Interestingly, the population from Jalisco can be readily identified by the high levels of the alkene C25:1 where it was the most abundant compound (above 40%) compared with all other populations where it was present at very low frequencies.

Geographical barriers, coupled with related ecological differences, may have driven population divergence and possibly explain the emergence of chemical differences and geographic chemotypes. In addition, orographic and biotopic particularities have probably acted selecting for particular CHCs to protect individuals under different environmental conditions, thus, favoring differentiation even further^47^. Generally, stingless bees show strong genetic structure^48–51^. This is a consequence of their particular mode of reproduction related to dependent colony foundation, in which a virgin queen swarms with a group of workers to start a new colony^52^. However, in contrast with other highly eusocial insects, newly formed colonies of stingless bees maintain long-time bonds for food and resources with the mother colony^25,53^. The link with the mother colony reduces the spreading of new colonies (and reproductive females) over long spaces. This favors genetic differentiation that is frequently associated with geographic distance in various species of stingless bees^47,48,54^.

Additionally, differences in chemical profiles may be one feature of the adaptation of populations to specific ecosystems, an essential aspect when considering colony trade. This implies that some species might suffer and perish due to intolerance to new climatic features^26^. In this regard, one practical aspect of our work is the identification of populations of two stingless bee species whose colonies are increasingly commercialized with a latent risk of being shipped to distant areas, many outside their original geographic region^27^. The effect of man induced translocation is evident by the weaker genetic structure of managed species of stingless bees compared to feral ones^51,55^. In Brazil for instance, the translocation of colonies poses a severe problem for species genetic integrity. At least 33 species of stingless bee have been negotiated and over 40% of sellers and buyers were located outside the natural range of the species^26,56^. Such trade has mixed populations and introduced species, allows the potential dissemination of hitchhiker symbionts and diseases, and mostly does not follow national legislation policies^26,56^. Interestingly, the results from the Mantel test indicate that *N. perilampoides* populations can be more clearly separated in accordance with the geographic region compared with *M. beecheii*. This could be an indication that populations of *N. perilampides* seem more structured, and probably no admixture has occurred as consequence of colony translocation for this species. In contrast, differences were less pronounced in accordance to geographic distance in *M. beecheii*. It may be possible that given the significance of this species for ancient stingless beekeeping, translocation of some colonies may have occurred in the past^25^. However, this deserves further analysis, possibly including other markers and more colonies, as it may also be an indication of recent admixture among populations. With the increasing popularity of stingless bees, in particular of *M. beecheii*, it would be important to monitor the movement of colonies across regions and CHCs could provide a rapid and reliable tool in conjunction with other methods^4,48,57^.

CHCs have high plasticity, allowing insects to quickly adapt their chemical profiles to external selection pressures^58^. Because of this, their use as taxonomic tools can be important in solving problems where other methods are not capable of detecting differences. In a pair of sibling species of orchid bees, DNA barcoding based on changes in the mitochondrial cytochrome c oxidase subunit 1 (COX1) showed no significant differentiation^59^, while microsatellite markers showed too much overlap in allele sizes to be diagnostic^60^. The species could nevertheless be distinguished based on their CHCs profiles^5^. Recently, geographic populations of *N. perilampoides*, showed no differentiation after the analyses of the nuclear regions ITS1, ITS2 nor the mitochondrial COX1. Differences were only observed for the mitochondrial 16S marker. However, different haplotypes differed only in one or two mutational steps, so in general, the nucleotide diversity was low for this marker^48^, rendering molecular tools of scarce utility for analyzing differentiation among populations of this species. However, as our study showed, significant differences could be found in the profile of CHCs of geographic populations of this species, rendering CHCs as a good set of epigenetic markers.

One important aspect for the use of CHCs as taxonomic tools is the genetic basis behind their variation among species^61^. In ants, recent studies have shown that cuticular profiles are heritable and species-specific^62,63^. In stingless bees, alkanes, alkenes and alkadienes showed no or little correlation with the geographical distribution of different species^64^. Similarly, chemical differentiation of thirteen species of *Melipona* bees showed no significant effect of environmental conditions^32^. These results indicate strong stability of CHC profiles over large geographical distances among species. Nevertheless, practically nothing is known about the relative contribution of environmental and genetic factors in determining intraspecific differentiation in stingless bees (and other social insects) making this is a pending and essential aspect to further study.

Our study found intraspecific CHCs variation in stingless bees which support their use as chemotaxonomic tools for the analysis of geographical differences within species. CHCs data bases could be included in automated systems in conjunction with other methods for an accurate identification of stingless bee populations and trace possible movements in the future. The relative influence of genetic and environmentally factors explaining CHC profile variation are still not clear and deserve further analysis. As CHCs are involved in several aspects of bee recognition and interactions, it would be key to unravel how these chemical signatures evolve across populations^45,65^.

### Supplementary Information

Supplementary Information.

## Data Availability

JJGQE is the corresponding author from whom materials can be requested.
