# Supplementary material for: Cuticular hydrocarbon profiles reveal geographic chemotypes in stingless bees (Hymenoptera: Meliponini)
Source: Sci Rep. 2024 Jun 24;14:14567. doi: 10.1038/s41598-024-65298-5 (PMC11196267; doi:10.1038/s41598-024-65298-5)
Supplement: Supplementary file 1 — Supplementary Information. [file 41598_2024_65298_MOESM1_ESM.docx]

| **Supplementary Table 1.** Retention times in minutes (RT) and Kovats indices, experimental = KI (exp) and from the literature = KI (Lit) from the different compounds found in *M. beecheii* and *N. perilampoides* (Tables 2 and 3). | | | |  |
| --- | --- | --- | --- | --- |
| **Compound** | **RT** | **KI (exp)** | **KI (Lit)** | |
| **Nonadecane** | 6.08 | 1899 | 1898^66^ | |
| **Heneicosane** | 7.01 | 2095 | 2098^66^ | |
| **Docosane** | 7.74 | 2193 | 2200^34^ | |
| **Tricosene** | 8.27 | 2268 | 2271^34^ | |
| **Tricosane** | 8.47 | 2291 | 2300^34^ | |
| **Tetracosane** | 9.53 | 2391 | 2400^34^ | |
| **Pentacosene 1** | 10.53 | 2469 | 2473^66^ | |
| **Pentacosene 2** | 10.61 | 2476 | 2473^66^ | |
| **Pentacosane** | 10.82 | 2492 | 2500^34^ | |
| **Hexacosane** | 12 | 2591 | 2600^34^ | |
| **Heptacosene 1** | 12.84 | 2669 | 2676^66^ | |
| **Heptacosene 2** | 12.92 | 2677 | 2683^66^ | |
| **Heptacosane** | 13.08 | 2691 | 2700^34^ | |
| **Octacosene** | 13.85 | 2771 | 2776^5^ | |
| **RT**= retention time in minutes, **KI (exp)**= experimental Kovats retention index, **KI (lit)**= Kovats retention index values found in literature. | | | |  |

| **(Cont.) Extended Data Table 1.** List of the cuticular hydrocarbons found in *M. beecheii* and *N. perilampoides* from different states and regions of Mexico. | | | |  |
| --- | --- | --- | --- | --- |
| **Compound** | **RT** | **KI (exp)** | **KI (lit)** | |
| **Octacosane** | 14.05 | 2791 | 2800^34^ | |
| **Nonacosene 1** | 14.8 | 2869 | 2880^66^ | |
| **Nonacosene 2** | 14.8 | 2879 | 2880^66^ | |
| **Nonacosane** | 14.9 | 2890 | 2900^34^ | |
| **Triacontane** | 15.8 | 2990 | 3000^34^ | |
| **Hentriacontene** | 16.4 | 3070 | 3076^66^ | |
| **Hentriacontane** | 16.6 | 3089 | 3100^34^ | |
| **Tritriacontene** | 18.3 | 3272 | 3278^5^ | |
| **Tritriacontane** | 18.4 | 3289 | 3300^34^ | |
| **RT**= retention time in minutes, **KI (exp)**= experimental Kovats retention index, **KI (lit)**= Kovats retention index values found in literature. | | | |  |

**References**

5. Pokorny, T., Lunau, K., Quezada-Euan, J. J. G. & Eltz, T. Cuticular hydrocarbons distinguish cryptic sibling species in *Euglossa* orchid bees. *Apidologie* **45**, 276–283 (2014).

34. NIST Mass Spectrometry Data Center. NIST Mass Spectral Search Program. Preprint at (2020).

66. Quezada-Euán, J. J. G. *et al.* Does sensory deception matter in eusocial obligate food robber systems? A study of *Lestrimelitta* and stingless bee hosts. *Anim Behav* **85**, 817–823 (2013).
